# Effects of Simultaneous Downregulation of PHD1 and Keap1 on Prevention and Reversal of Liver Fibrosis in Mice

**DOI:** 10.3389/fphar.2018.00555

**Published:** 2018-05-30

**Authors:** Jing Liu, Wencai Li, Manoj H. Limbu, Yiping Li, Zhi Wang, Zhengyuan Cheng, Xiaoyi Zhang, Pingsheng Chen

**Affiliations:** ^1^Department of Pathology, The First Affiliated Hospital of Zhengzhou University, Zhengzhou, China; ^2^Department of Pathology and Pathophysiology, School of Medicine, Southeast University, Nanjing, China

**Keywords:** liver fibrosis, hepatocytes, PHD1, Keap1, hypoxia, oxidative stress

## Abstract

**Background and Aim:** To investigate whether double-knockdown of PHD1 and Keap1 in mice could enhance the resolution of carbon tetrachloride (CCl_4_)-induced liver fibrosis.

**Methods:** The liver fibrosis model of mice was established by intraperitoneal injection of 25% CCl_4_ in olive oil (4 ul/g) twice a week for 8 weeks. PHD1shRNA and Keap1shRNA eukaryotic expression plasmids were simultaneously administered from the beginning of the first to fourth week (preventive group) or from the fifth to eighth week of CCl_4_ injection (therapeutic group) via hydrodynamic-based tail vein injection. Successful transfection was confirmed with the expression of red fluorescent protein and green fluorescent protein in hepatocytes. Western blot was used for determining the expression of PHD1 and Keap1, HE, Sirius red, and Masson staining for evaluating the histopathological stages of fibrosis. Immunohistochemical techniques were applied to evaluate the expression of a-SMA.

**Results:** The fluorescence of red and green were observed mainly in hepatocytes, and downregulation of PHD1 and Keap1 expression in liver was detected by western blot. Meanwhile, double-knockdown of PHD1 and Keap1 in mice alleviated liver fibrosis, and the effect was further enhanced especially in the preventive group. Immunocytochemical staining showed decreased expression of a-SMA when both PHD1 and Keap1 were knockdown.

**Conclusion:** Downregulation of PHD1 and Keap1 expression in the liver could be achieved via hydrodynamic injection of PHD1shRNA and Keap1shRNA, thereby, preventing liver fibrosis.

## Introduction

Liver fibrosis is a type of liver scarring from compensatory response to various chronic liver injuries resulting in disruption of extracellular matrix, cell populations, and cytokines. As a metabolically active organ, liver is extremely sensitive to hypoxia and hypoxia in liver fibrosis is mainly due to sinusoidal stenosis, neovascularization, collagen deposition, and abnormal hepatic vascular network. It has been postulated that liver injury are often accompanied by hypoxia (Onori et al., 2000). In recent years, studies have shown that oxidative stress plays an important role in the development of liver fibrosis (Yang et al., 2013) and often factors involved in liver fibrosis are accompanied by increased oxidation or decreased antioxidant. The inter relationship between oxidative stress and liver fibrosis are currently among the hot topic of research. PHD1 and Keap1 are the intracellular oxygen sensor and oxidative stress sensor respectively, and we have used the *in vitro* shRNA technology to interfere with their functions in the hepatocytes, and thereby reducing hepatocytes hypoxia and oxidative stress (Liu et al., 2017). In this study, PHD1shRNA and Keap1shRNA plasmids were simultaneously transfected into the mice with liver fibrosis via hydrodynamic injection, and the subsequent effects of PHD1 and Keap1 on the prevention and reversal of liver fibrosis were observed.

## Materials and Methods

### PHD1shRNA and Keap1shRNA Plasmid Construction

The plasmids pGPU6/RFP/Neo-PHD1shRNA and pGPU6/RFP/Neo-PHD1shNC were both designed by GenePharma (Shanghai, China). For silencing Keap1 gene expression, the plasmid Plvx/GFP/puro-Keap1shRNA was designed by Hanbio Biotechnology (Shanghai, China). The purity and quantity of the DNA were measured by spectrophotometry (Thermo, United States) at 260/280 nm.

### Establishment of CCl_4_-Induced Liver Fibrosis Model

Sixty healthy male ICR mice, 6 weeks old, each weighing about 16–20 g were purchased from Yangzhou University Animal Experiment Center. Mice were housed at temperature of 20–28°C, relative humidity of 60–70% with normal ventilation and 12 h light and dark cycle. During whole period of experiment, mice were provided free access to sterile deionized water and standard normal food. This study was carried out in accordance with the recommendations of the European Council Directive of the 24^*th*^ November 1986 (86/609/EEC). All procedures were approved by the Animal Research Ethics Committee at the Medical School of the Southeast University (Nanjing, China).

Mice were divided into the following six groups, and 10 in each group: (1) Blank control group were administered normal saline, twice a week for 8 weeks. (2) CCl_4_ model group were administered 5 μL/g of 25% CCl_4_ in olive oil, twice a week for 8 weeks. (3) PHD1shNC group were treated CCl_4_ similar to model group for 8 weeks along with PHD1shNC empty plasmid hydrodynamic injection once a week from week 1 to week 4. (4) Single transfection group (PHD1shRNA group) were treated same as model group along with PHD1shRNA injection once weekly from week 1 to week 4 (5) Co-transfection preventive group was similar to model group along with co-injection of PHD1shRNA and Keap1shRNA once weekly from week 1 to week 4. (6) Co-transfection therapeutic group were treated similar to model group along with co-injection of PHD1shRNA and Keap1shRNA plasmid once weekly from week 4 to week 8.

After 8 weeks, mice were killed with intraperitoneally injection of pentobarbital solution. Livers were dissected and washed in PBS and photographs photographed. At the same time, heart, spleen, lung, kidney, and other organs were preserved for the follow-up experiments. Half of tissues were fixed in a 10% formalin. Part of the tissues were embedded in OCT (optimal cutting temperature compound) for frozen sections, and the rest were snap frozen in liquid nitrogen and stored at -80°C for WB.

### Hydrodynamic Injection of PHD1shRNA and Keap1 shRNA Plasmids

Hundred μg each of PHD1shRNA and Keap1shRNA plasmid DNA were mixed in a 2 mL solution of 0.9% sodium chloride and was injected through the tail vein of mice within 6 to 7 s, the frequency of injection was once a week for 4 weeks (Maruyama et al., 2002; Chen et al., 2004; Zender et al., 2005). The mice were sacrificed 24 h after the last injection, and frozen sections were prepared from the tissues of liver, heart, spleen, lung, and kidney and then washed in a cold acetone for 10 min. After being allowed to dry, the cells were washed in PBS for 5 min for three times, and DAPI (4′,6-diamidino-2-phenylindole) staining for 5 min was done next. Finally, the sections were observed under fluorescence microscope for the expression of RFP (red fluorescence protein) and GFP (green fluorescence protein) for determining if the transfection was successful. The protein expression levels of PHD1 and Keap1 *in vivo* were detected by WB using a polyclonal antibody to PHD1 (Abcam, United States) and Keap1 (Proteintech, United States).

### Body Weight and Liver Index

Body weight were recorded before each intraperitoneal injection of CCl_4_ and were also calculated after 8 weeks. Liver index was calculated according to the following formula: liver index = wet weight of liver/body weight × 100%.

### Liver Function

Intraocular venous blood was collected and centrifuged at 3000 rpm/min for 5 min. The supernatants were collected and measured for ALT, AST, and ALP levels by automatic blood biochemical analyzer.

### Liver Histology and Immunohistochemical Staining

The sections were cut into 3–5 μm thick slices with a microtome, and stained with hematoxylin and eosin (H&E), Masson dyes, and sirius red.

According to Scheuer’s program (Scheuer, 1991), liver fibrosis were graded into S0∼S4 stages, S0: no fibrosis. S1: the portal area, periportal fibrosis, limiting plate fibrosis or intralobular fibrosis, neither of which affects the structural integrity of the lobules. S2: firbous septum formation due to bridging necrosis but lobular structure is preserved. S3: a large number of fibrous septum dividing hepatic lobules, causing distortion of lobular structure, but no cirrhosis formation. S4: early cirrhosis, extensive destruction of liver parenchyma, diffuse fibrogenesis, and pseudolobule formation. The semiquantitative scoring system (SSS) for liver fibrosis was done according to the Cheveallier’s protocol (Chevallier et al., 1994).

Next, the expression of a-SMA in liver tissue was detected by immunohistochemistry. Paraffin sections were antigen-retrieved, blocked with 5% BSA, and then was incubated with primary antibody a-SMA (Proteintech, United States) for overnight. Next, section were incubated with secondary antibody for 20 min at 37°C, then coloration with 3, 3-Diaminobenzidine (DAB) was done, next, the nuclei were counterstained with hematoxylin, and then were observed under the light microscope and subjected to semi-quantitative analysis using Image J software.

### Statistical Analysis

All the results are expressed as mean ± SD. One-way analysis of variance (ANOVA) and the Student’s *t*-test were used to evaluate the difference between groups. SPSS16.0 software was used for statistical analysis of data. A *p*-value < 0.05 was considered significant.

## Results

### Fluorescence Observation of Each Organ Sections

**FIGURE 1:**
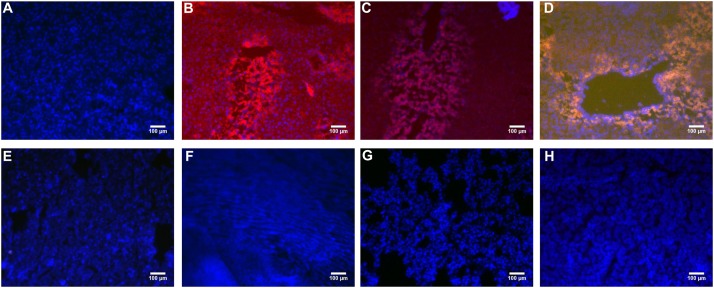
The PHD1shRNA, PHD1shNC, PHD1+Keap1shRNAs expression vectors were injected into CCl_4_-treated mice, respectively. The fluorescence from the fibrotic liver of blank control group **(A)**, PHD1shRNA group **(B)**, PHD1shNC group **(C)**, PHD1+Keap1shRNAs-transfected group **(D)**, and from Heart **(E)**, Lung **(F)**, Spleen **(G)**, Kidney **(H)** of PHD1+Keap1shRNAs-transfected group were observed by fluorescent microscope. DAPI staining for cell nuclei in the same field.

PHD1shRNA and Keap1shRNA plasmids were injected into mice via tail vein and then observed by fluorescence microscopy (**Figure 1**). Obvious red and green fluorescence were observed in liver tissues. DAPI staining of nuclei suggested that most of the fluorescence existed within the hepatocytes. The fluorescence in other organs were weak and some were even undetectable. Thus, successful targeted transfection of hepatocytes were achieved using hydrodynamic injection.

### PHD1 and Keap1 Protein Expression

**FIGURE 2:**
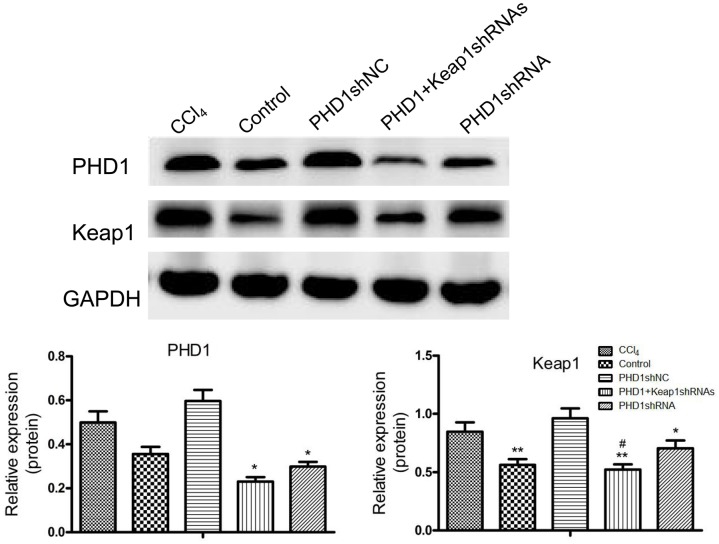
The expressions of PHD1 and Keap1 in fibrotic liver tissues were assayed by western blot. GAPDH was used as the internal standard, each value represents mean ± SD (*n* = 3). ^∗^*p* < 0.05, ^∗∗^*p* < 0.01 vs. CCl_4_ group, ^#^*p* < 0.05 vs. PHD1shRNA group.

PHD1shRNA and Keap1shRNA plasmids were successfully targeted to the hepatocytes via tail vein, and expression of PHD1 and Keap1 were detected by using western blot (**Figure 2**). Compared with that in the blank control group, the expression of Keap1 in the model group was increased significantly, however, the expression of PHD1 only changed by little. There were no significant differences in the PHD1 and Keap1 levels between PHD1shNC group and the model group (*p* > 0.05), and we found that the levels of PHD1 and Keap1 in the single transfection group were decreased, while those in the co-transfection group were further reduced.

### Mouse Liver Morphology

**FIGURE 3:**
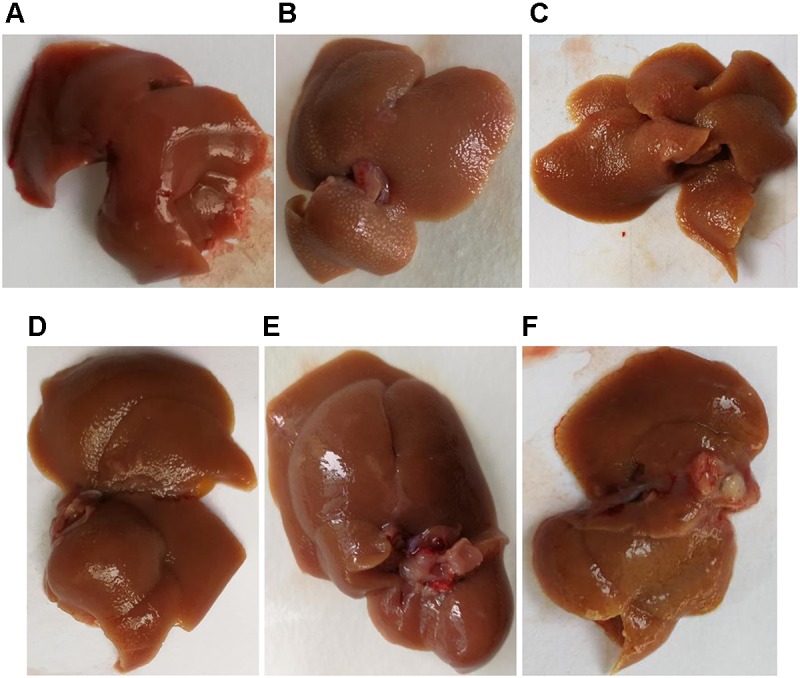
Liver morphology of different group, **(A)** blank control group, **(B)** CCl_4_-induced liver fibrosis group, **(C)** PHD1shNC group, **(D)** PHD1shRNA group, **(E)** PHD1+Keap1shRNAs-transfected prevention group, and **(F)** therapeutic group.

The **Figure 3** showed that the liver in the blank group was smooth, ruddy and soft textured, while the model group had obvious adhesion with the surrounding tissues, the edges were blunt and the surface were granular, the changes in the PHD1shNC group were similar to that of the model group. The liver from the co-transfection preventive group showed no obvious changes, even though the color was not as ruddy as that of blank group, the lesions were less severe than the model group. The pathological changes in the single-transfection group and co-transfection therapeutic group were little more severe than in the preventive group.

### Mice Weight and Liver Index

As shown in **Table 1**, the model group had weight reduction (*p* < 0.01) than the blank control group. There were no significant differences in the weight between the single transfection group and the co-transfection preventive group, but both had increased weight when compared to the model group (*p* < 0.05). However, the weight of the co-transfection therapeutic group was slightly higher than those of the model group, but was not statistically significant (*p* > 0.05). Compared to the blank group, the liver index of model group increased significantly (*p* < 0.05), however, there were no significant differences between the PHDshNC group and the model group, and there was also no significant difference between co-transfection group and single transfection group (*p* > 0.05). The liver index in the co-transfection preventive group were lower than those in the model group (*p* < 0.05), but the single transfection group and the co-transfection therapeutic group did not reach statistical significance when compared to the model group (*p* > 0.05).

**Table 1:** The weight and liver index in different groups.

Group	Weight		Liver index (%)
	0 week	8 week	
Blank control	27.83 ± 1.52	43.25 ± 1.06	4.27 ± 0.62
CCl_4_	27.66 ± 1.25	35.05 ± 0.91^ 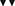 ^	7.70 ± 1.45^ 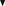 ^
PHD1shNC	27.67 ± 1.75	36.05 ± 0.97	6.60 ± 1.02
PHD1shRNA	27.5 ± 1.80	37.95 ± 0.89^∗^	6.10 ± 0.94
PHD1+Keap1shRNAs co-transfection preventive group	27.5 ± 2.29	39.02 ± 1.79^∗^	5.10 ± 0.60^∗^
PHD1+Keap1shRNAs co-transfection therapeutic group	27.66 ± 2.25	36.72 ± 1.19	6.20 ± 0.83

*^∗^p < 0.05 vs. CCl_4_, ^
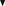
^p < 0.01, ^
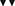
^p < 0.001 vs. blank control.*

### Liver Functions

As shown in **Table 2**, liver functions test showed that when compared to the blank group, the levels of serum ALT and AST in the model group were significantly increased, while the levels in the PHDshNC group were not significantly different from those in the model group (*p* > 0.05). The levels in the single transfection group were decreased than that in the model group, while the decrease in the co-transfection preventive group were more obvious (statistically significant when compared to single transfection group), which indicated that co-inhibition of PHD1 and Keap1 expression could better prevent liver injury in mice. However, the reduction in the co-transfection therapeutic group was not as effective as that of preventive group. The level of ALP in the model group was higher than that in the blank control group and the ALP in the co-transfection preventive group was lower than that in the model group, but the ALP levels of all six groups were within the normal range.

**Table 2:** The ALT, AST, ALP levels of different groups.

Group	ALT	AST	ALP
Blank control	39 ± 3.94	36 ± 3.28	38 ± 6.16
CCl_4_	436 ± 61.15^ 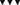 ^	316 ± 48.18^ 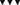 ^	62 ± 5.66^ 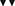 ^
PHD1shNC	368 ± 45.44	262 ± 42.68	52 ± 4.99
PHD1shRNA	222 ± 22.05^∗∗^	99 ± 12.85^∗∗^	56 ± 5.22
PHD1+Keap1shRNAs co-transfection preventive group	145 ± 16.98^∗∗##^	71 ± 7.6^∗∗∗#^	42 ± 4.57^∗∗^
PHD1+Keap1shRNAs co-transfection therapeutic group	193 ± 24.86^∗∗^	78 ± 11.23^∗∗^	60 ± 7.66

*^∗^p < 0.05, ^∗∗^p < 0.01, ^∗∗∗^p < 0.001 vs. CCl_4_, ^#^p < 0.05, ^##^p < 0.01 vs. PHD1shRNA, ^
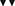
^p < 0.01, ^
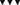
^p < 0.001 vs. blank control.*

### Liver Fibrosis Grades and Scores

**FIGURE 4:**
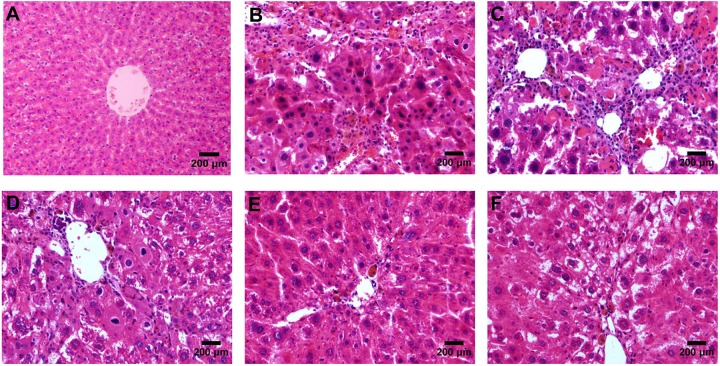
HE staining of liver tissues, **(A)** blank control group, **(B)** the CCl_4_-induced liver fibrosis group, **(C)** PHD1shNC group, **(D)** PHD1shRNA group, **(E)** PHD1+Keap1shRNAs co-transfected prevention group, and **(F)** treatment group.

**FIGURE 5:**
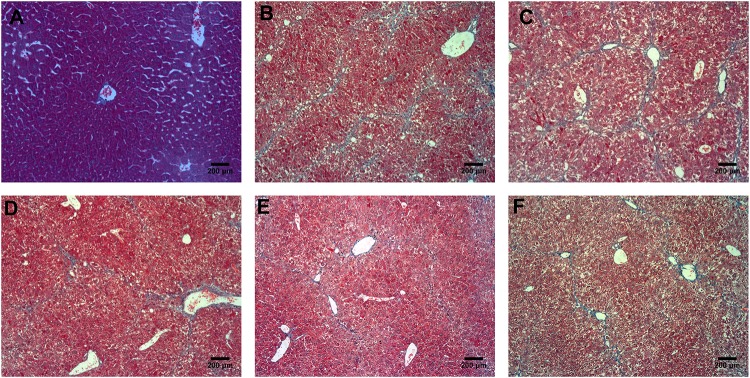
Masson staining of liver tissues, **(A)** normal control group, **(B)** the CCl_4_-induced liver fibrosis group, **(C)** PHD1shNC group, **(D)** PHD1shRNA group, **(E)** PHD1+Keap1shRNAs co-transfected prevention group, and **(F)** treatment group.

**FIGURE 6:**
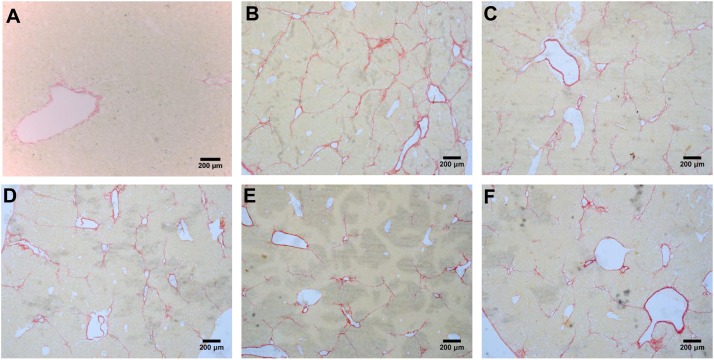
Sirius red staining of liver tissues, **(A)** blank control group, **(B)** the CCl_4_-induced liver fibrosis group, **(C)** PHD1shNC group, **(D)** PHD1shRNA group, **(E)** PHD1+Keap1shRNAs co-transfected prevention group, and **(F)** treatment group.

According to the results of HE (**Figure 4**), Masson (**Figure 5**) and Sirius red staining (**Figure 6**), the degree of liver fibrosis was graded and scored for each group in **Table 3**. The liver fibrosis stage in model group and PHDshNC group were mostly S3∼S4 phases, and the single transfection group were mostly S1∼S2 phases. Most of the co-transfection preventive groups were S1 phase while most of the co-transfection therapeutic group were S2∼S3 phases. Scores between PHD1shNC group and the model group had no significant differences, while scores of single transfection group, co-transfection preventive group and co-transfection therapeutic group were lower than that of model group. Whereas, scores of co-transfection therapeutic group was higher than in the co-transfection preventive group.

**Table 3:** The grades and scores of liver fibrosis in different groups.

Group	Quantity	Staging	Scoring
Blank control group	10	S0	0
CCl_4_ model group	10	S3∼S4	20.6 ± 3.78
PHD1shNC empty plasmid group	10	S3∼S4	18.7 ± 3.43
PHD1 shRNA group	10	Mostly S1∼S2, little for the S3	9.8 ± 1.79^∗^
PHD1+Keap1shRNAs co-transfection preventive group	10	Mostly S1, little for the S2	6.5 ± 1.19^∗∗#^
PHD1+Keap1shRNAs co-transfection therapeutic group	10	S2∼S3	11.5 ± 2.11^∗^

*^∗^p < 0.05, ^∗∗^p < 0.01 vs. CCl_4_, ^#^p < 0.05 vs. PHD1shRNA.*

### a-SMA Expression in Liver Tissue

**FIGURE 7:**
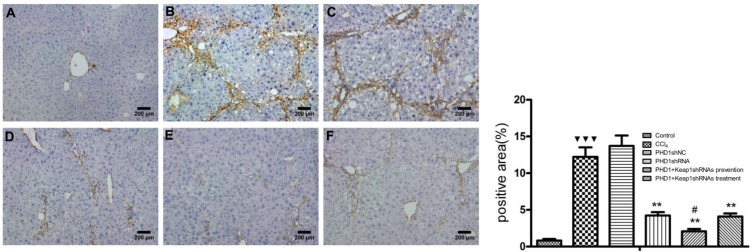
The expression of a-SMA were measured by immunohistochemical staining. **(A)** blank control group, **(B)** the CCl_4_-induced liver fibrosis group, **(C)** PHD1shNC group, **(D)** PHD1shRNA group, **(E)** PHD1+Keap1shRNAs co-transfected preventive group, and **(F)** therapeutic group. Each value represents mean ± SD (*n* = 3). ^∗∗^*p* < 0.01 vs. CCl_4_ group, ^#^*p* < 0.05 vs. PHD1shRNA group, ^
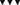
^*p* < 0.001 vs. control.

a-SMA is an indicator of HSCs activation. In normal liver, a-SMA was mainly expressed on the walls of central vein, periportal, and outside the vessel walls. In the model group, a-SMA was strongly expressed in the fibrous septa and perivascular areas, while there were no significant differences between the PHDshNC group and model group (*p* > 0.05) (**Figure 7**). Compared to the model group, a-SMA expression in the co-transfection preventive group was weaker (*p* < 0.01) and mainly around the incomplete fibrous speta. In addition, the expression of a-SMA in co-transfection therapeutic group was significantly decreased. Therefore, the above results indicates that the activation of HSCs can specifically be inhibited by downregulating the expression of PHD1 and Keap1 in rats with hepatic fibrosis.

## Discussion

Liver fibrosis is a pathological process caused by chronic liver injury. There are many factors such as viral hepatitis, alcohol and drugs which results in chronic liver injury (Begriche et al., 2011). CCl_4_ is a hepatotoxin widely used for establishing animal models of liver fibrosis. It acts on hepatocytes directly by causing tissue necrosis. In this study, the injection of 25% CCl_4_ in olive oil at the dose of 5 μL/g for 8 weeks resulted in severe liver injury in the model group. The liver histology of model group showed disruption of normal hepatic architecture, infiltration of inflammatory cells, necrosis, broad fibrous septa with pseudolobules. Thus, it concluded that CCl_4_ induced hepatic fibrosis was successfully established.

In recent years, gene therapy has become a hot topic of research in the treatment of liver fibrosis. RNAi is one of the most widely studied techniques in gene therapy, and shRNA has been widely used *in vivo* experiment for its high efficiency and strong stability. However, owing to the complexity of the human body and lack of specific target, it leads to adverse effects. Therefore, the key to the treatment of liver fibrosis would be to design an effective and specific targeted gene delivery system. Currently, the effective vectors for gene delivery are mainly divided into viral vectors and non-viral vectors. However, viral vectors have some limitations in clinical application due to their immunogenicity and carcinogenic properties. On the other hand, non-viral vectors in the recent years has been studied extensively with considerable progress in targeted anti-fibrotic approach in the treatment of liver fibrosis. Asialoglycoprotein receptor is an efficient endocytic vector present in the liver parenchymal cells and it is widely used in targeted drug therapy of liver disease. Sato et al. (2007) reported that by injecting galactosylated liposomes encapsulated siRNA into the tail vein of mice can achieve an effective gene silencing in the liver cells of those mice. However, it has been reported that cationic liposome/DNA complexes injection through the tail vein are associated with certain degree of hepatotoxicity (Tousignant et al., 2000; Loisel et al., 2001; Kawakami et al., 2006). Moreover, during the study of targeted drugs via receptor-mediation, the receptor density and its binding activity vector/drug interaction and vector/internal environment interactions must also be put into consideration. It should also be considered that low transport efficiency of non-viral vectors is the biggest bottleneck preventing its widespread application.

Hydrodynamic injection is the solution for the above mentioned shortcoming of non viral vectors (Song et al., 2002), which was discovered by Liu et al. (1999) and Zhang et al. (1999). Dynamic CT scans monitoring has already confirmed that the hydrodynamic injection could achieve better liver targeting effects (Yokoo et al., 2015). Hydrodynamic injection of siRNA or shRNA naked plasmid could inhibit the genes expression in the liver (McCaffrey et al., 2002; Song et al., 2003), Abe et al. (2016) also has recently found that by mediating MMP-13 expression via hydrodynamic injection could effectively inhibit liver fibrosis in rats. In addition, hydrodynamic injection of HbeAg-negative cccDNA into C57BL/6J mice resulted in a nearly 100% HBV infection in the initial stage (Wang et al., 2017). Clinical trials in human has also shown that hydrodynamic injection of thrombopoietin plasmids through human portal vein could markedly increase platelet count (Khorsandi et al., 2008).

It is believed that the rapid injection of large volume of plasmid solution leads to the transient right heart failure which results into a temporary systemic circulatory congestions, and increasing the contact time between the DNA and intrahepatic cells. Moreover, rapid injection of the DNA also minimizes its rapid degradation in the blood, thereby allowing prolong contact of the DNAs with the cells (Maruyama et al., 2002). Pressure also has the key role in allowing the liver cells to absorb DNA plasmids. Speed and volume of injection also directly affects the efficiency of DNA transfection, therefore, slow rate of injection and reduced volume reduces the efficiency of gene expression. Studies have shown that injecting the DNA volume of about 8% of total body weight within 7 s could achieve maximum expression level (Liu et al., 1999; Zhang et al., 1999). However, due to the limitation of animal tolerance, the volume of DNA injected hits plateau after the volume of 100 μg (Zhang et al., 1999). Moreover, a study has found that the expression of exogenous genes in the liver peaks within 8–24 h after hydrodynamic injections and decreases significantly within 6 days, but higher levels can still be detected. The safety and efficacy of hydrodynamic injection has also been reported by a large number of researchers in recent years (Kamimura et al., 2009, 2013, 2014), and although hydrodynamic injection has been reported to cause a slight increase in ALT, it is transient and regresses to normal range within days (Suda et al., 2008; Yokoo et al., 2013).

Based on hydrodynamic injection technique, in this experiment, plasmid solution was injected at the concentration of 100 μg/2 mL within 6 s, and once every 5 days. The results showed that hydrodynamic injection resulted in high expression of RFP and GFP only in the liver but not in other tissues such as heart, spleen, lung, and kidney. Western blot also showed good targeted interference effect in the liver tissues. Compared to single transfection group, co-transfection group could better reduce PHD1 and Keap1 protein levels, and were consistent with the results of our *in vitro* experiments (Liu et al., 2017).

The results of liver function tests and histopathology showed that the anti-fibrotic effect in the co-transfection group was significantly better than that in the single transfection group, while there were no significant difference between PHD1shNC group and CCl_4_ model group. In addition, the degree of hepatocyte injury and fibrosis in mice from both preventive group and therapeutic group were lesser than the model group, but the preventive group showed significantly reduced liver fibrosis damage than the treatment group. Thus, this indicates that interfering with the expression of PHD1 and Keap1 in the hepatocytes has obvious preventive effect against the CCl_4_-induced hepatic fibrosis.

Damaged hepatocytes releases various cytokines and activates HSCs which is marked by expression of a-SMA. Therefore, inhibiting HSCs activation could be the key to anti fibrotic process (Yu et al., 2012; Su et al., 2013). Our study found that a-SMA expression in the co-transfection preventive group was relatively decreased than in the single transfection group and also lower than in the therapeutic group. Therefore, it shows that the interference of PHD1 and Keap1 in hepatocytes *in vivo* could indirectly inhibit the activation of HSCs, and could synergisticly effect each other.

PHD1shRNA and Keap1shRNA plasmids injections via hydrodynamic injection could offer a novel strategy in the prophylaxis of liver fibrosis, however, there are still some shortcomings such as difficulty in its operation and discrepancies in its effects among individuals. Therefore, further detailed study about this method is necessary. Since fibrosis could still be seen in the co-transfection preventive group, which suggests that co-transfection does not completely prevent fibrosis. As liver fibrosis is a complex process involving different types of cells, cytokines and signaling pathways, and it may also cross react with other genes while interfering with PHD1 and Keap1 genes. Therefore, it is imperative to develop safe and combined multi-gene and multi-factor targeted therapy.

## Author Contributions

JL, YL, and PC: conceived and designed the experiments. JL, ML, ZW, ZC, and XZ: performed the experiments. JL and YL: analyzed the data and wrote the paper. YL, WL, and PC: revised the paper.

## Conflict of Interest Statement

The authors declare that the research was conducted in the absence of any commercial or financial relationships that could be construed as a potential conflict of interest.
